# Considerations for dual barrel electrode fabrication and experimentation†

**DOI:** 10.1039/d3an01969a

**Published:** 2024-01-23

**Authors:** Lynn E. Krushinski, Philip J. Kauffmann, Amber K. Wang, Jeffrey E. Dick

**Affiliations:** a Department of Chemistry, Purdue University West Lafayette IN 47907 USA jdick@purdue.edu; b Elmore Family School of Electrical and Computer Engineering, Purdue University West Lafayette IN 47907 USA

## Abstract

New electrochemical probes offer the opportunity to investigate new systems. A dual barrel electrode can be laser pulled to produce micron-sized platinum disk electrodes. Here, we detail several important considerations for both the fabrication process and for experimental implimentation of the probe. We provide parameters for a Sutter P-2000 laser puller, methods for optical and electrochemical characterization, tips for how to successfully bevel the microelectrodes, and how salt concentrations and electrostatic discharge affect the voltammetry. This paper serves as a guide for how to successfully implement dual barrel electrodes from fabrication to experimentation.

## Abstract 2

New electrochemical probes offer the opportunity to investigate new systems. In this work, we provide a guide for how to fabricate dual barrel, Pt disk electrodes. We include data and procedures for characterizing and troubleshooting the electrodes.

## Graphical abstract

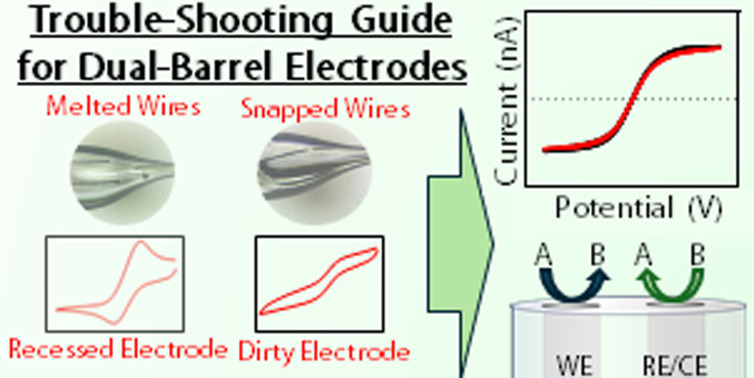

## Introduction

Electrochemistry is a sensitive and versatile tool for investigating many natural phenomena. Often, an electrochemical cell uses three distinct probes as the working, reference, and counter electrodes. Depending on the experimental design, the setup can vary widely. For example, there are numerous different types of working electrodes^1–4^ and reference electrodes.^5–7^ In other situations, the size of the electrodes will vary such as when smaller environments necessitate the need for micrometer^8,9^ to nanometer^10–12^ sized working electrodes. However, even with a nanometer sized working electrode, the separate reference and counter electrode take up significant space. Recent reports have shown the fabrication and application of multi-barrel electrodes to miniaturize the electrochemical cell into a single probe. Filotás *et al.* created a triple-barrel electrode to study galvanic corrosion processes.^13^ Our group has also created a triple-barrel electrode capable of electroanalysis for small droplets, single-cells, and aerosols.^14^

Still, these single-capillary, multi-barrel electrodes can be simplified further because two electrodes are the minimum requirement to make an electrochemical measurement. Many kinds of electrochemical cells using dual-barrel probes have been reported. Tomita and Wardell reported early on the coupling resistance of a double-barreled microelectrode.^15^ More recently, the Unwin group introduced scanning electrochemical cell microscopy by using a theta-barrel pipet to simultaneously measure material topographical and functional properties,^16^ and they lay out in detail how to fabricate carbon dual-barrel electrodes for the application of SECM.^17^ Neither of these electrodes make use of platinum disk electrodes. Filotás *et al.* designed a dual-barrel probe that incorporated the reference electrode into the capillary to investigate the corrosion of surfaces.^18^ This electrode was fabricated with a filament heater, gravity-assisted puller. These examples highlight the diverse types of dual-barrel electrodes or pipettes available to the scientific community, as the fabrication and applications of these probes vary greatly.

Here, we present a troubleshooting guide for dual-barrel, platinum disk electrodes fabricated with a laser-assisted puller. The materials and methods presented here are optimal for creating a single probe capable of carrying out electrochemical measurements. Our electrode uses the first disk as the working electrode and the second disk as the quasi-reference/counter electrode. While a laser-pulled Pt disk dual-barrel electrode has been previously reported,^19^ it does not provide an extensive trouble-shooting and fabrication guide. These types of probes are important because they can be used in generation-collection experiments, and they offer the opportunity to electrochemically probe the liquid|air interface.^20,21^ Our lab has previously reported the use of these specific dual-barrel electrodes for probing single aerosols^20^ and single acoustically levitated droplets.^21^ Thus, we provide a detailed report on the fabrication of these probes, trouble-shooting solutions for common errors encountered while using a laser-puller, as well as several experimental considerations when using this type of probe.

## Results and discussion

### Fabrication of dual barrel electrodes

**Fig. 1:**
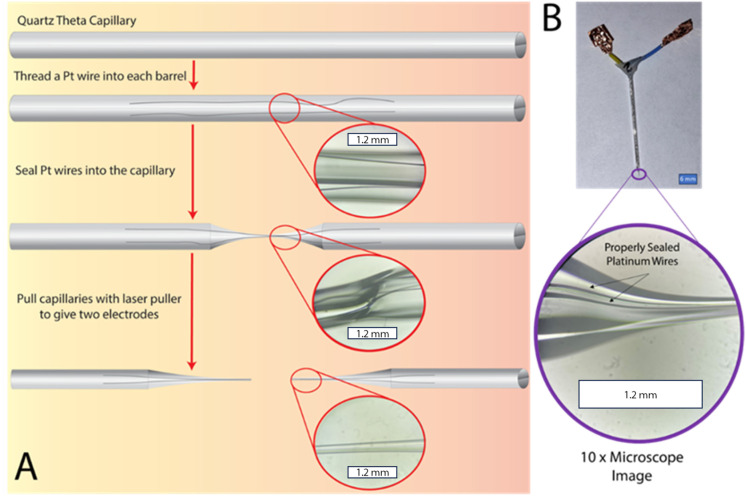
(A) Schematic of dual barrel electrode (DBE) fabrication process. (B) Photograph of a fabricated DBE with a microscope image of the proper seal. All inserted images were taken with a 10× objective.

Dual barrel electrodes (DBEs), and multi-barrel electrodes in general, are most commonly fabricated using an electrode/micropipette puller which contains some type of central heating element (such as a heated filament or laser) where a capillary (typically quartz or borosilicate) threaded with metal wire (typically platinum or gold) is heated until a seal is achieved and subsequently pulled.^18,22–25^ The ideal result of this process is two identical capillaries with a fine taper at the end and exposed inlaid disk electrodes with diameters ranging from 10s of μm to 100s of nm. A schematic showing a DBE through the typical fabrication process with inset microscope images can be seen in Fig. 1. To fabricate a functional DBE, the parameters used to seal and pull these electrodes must be optimized. As mentioned in our previous work,^26^ the optimization may look slightly different between instruments. Additionally, our experience has shown that these parameters may need to be adjusted over time. Thus, our suggested parameters can be found in Table 1 as a starting point for laser-pulling dual barrel electrodes.

**Table 1:** Suggested starting parameters for the P-2000 laser puller with dual barrel, Pt wire electrodes

Step	Heat	Filament	Velocity	Delay	Pull
Seal	750	5	100	200	0
	30 s ON/OFF ×4 cycles, flip capillary, ×4 cycles				
Pull	725	3	120	128	250
	Heat on time ∼5 s				

Herein, we focus solely on platinum DBE fabrication using a Sutter P-2000 laser puller. A more specific step-by-step guide with pictures and labels for this specific model of laser puller can be found in Fig. S1.† While these parameters and procedures were optimized for platinum wire, the same principles outlined below can be used for the fabrication of other electrode types (such as gold). Regardless of the specific puller or metal wire used, the goal of the DBE pulling process is two Pt wires completely and continuously sealed within the capillary.

To begin, a threaded capillary is first centrally placed in the laser puller (and kept in place by placing an insert to prevent the pull action) and connected to vacuum to prevent air bubbles from being trapped within the sealed portion of the capillary. Then the center of the capillary is heated until it has reformed to encase and tightly seal the threaded wire. For DBEs, a quartz theta capillary was used to ensure both wires remain separated during the fabrication process. We have noticed that for these capillaries in particular, a proper seal is more easily obtainable when the capillary is rotated 180 degrees halfway through the sealing process. We have also noticed that these capillaries require more sealing steps in general as compared to typical single barrel capillaries.^26^ A cyclic process is ideal as it allows the Pt wire to cool between heat applications, avoiding the common error of melting the wires and rendering them unable to be pulled. In our experience, cycles of 30–40 s of the laser on followed by the same amount of time for the laser off works well. Importantly, the time the laser is off should equal however long the laser is on in a cycle, so that the capillary and Pt wires do not overheat and melt. During this process, the seal can be checked under a standing microscope before the pulling step to ensure a proper seal has been achieved. After the sealing step is complete, the vacuum and the inserts are removed from the capillary and the pulling step is run. The pulled electrodes can then be checked under a standing microscope. Once a proper seal of the pulled electrode is verified, the electrodes can either be exposed through polishing on a nanopipette beveller or by cutting the tip of the electrode with scissors or a razor blade. An example of a fabricated DBE with a proper glass seal around continuous platinum wires can be seen in Fig. 1B.

**Fig. 2:**
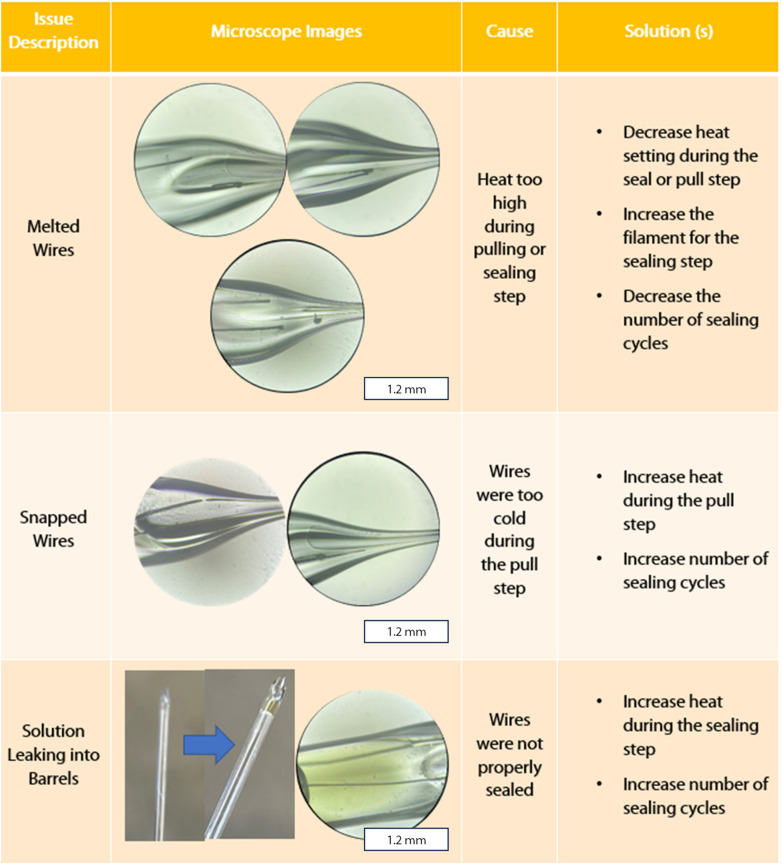
DBE pulling troubleshooting guide with microscopy. All inset images microscope images were taken with a 10× objective.

There are several challenges in optimizing the fabrication of DBEs. A troubleshooting guide with example microscope images of the most common problems we encountered, as well as proposed solutions, can be found in Fig. 2. These issues can arise during the sealing and pulling steps, such as an incomplete seal after the sealing step, or the sealed wires may melt or snap during the pulling step. The first (and perhaps most common) challenge is avoiding melting wires. This occurs as a result of the heat being too high during either the sealing or pull steps. To diagnose which step is responsible, one can check the electrode between the sealing and pulling step. To prevent melting, the heat setting for the laser can be decreased at either or both the sealing and pull step. We recommend that the temperature is changed in small increments (we suggest 5 at a time). Lower temperature settings with an increased number of sealing cycles may also help prevent the wire from melting, at the cost of increased fabrication time per electrode. On the other hand, temperature settings that are too low will result in either unsealed or snapped wires after the sealing and pulling steps, respectively. If the sealing process required more than 10 cycles overall, or there is no noticeable increase or change in the seal between cycles, the heat should be increased incrementally (we recommend 5 points at a time). We found that a procedure of 4 cycles of 30 s ON/OFF, rotating the capillary 180 degrees, then 4 cycles of 30 s ON/OFF should be enough to seal the Pt wires when the heat is sufficiently high. If the wire shows a clean break after the pulling process, the heat can be increased by 5–10 points at a time until the wire is pulled smoothly and continuously. Finally, the solution used for testing the DBE can sometimes enter the capillary if the seal is incomplete or if the DBE has been beveled down below the portion of sealed wire. In this case, the DBE will not be usable and will need to be remade.

### Characterization of dual barrel electrodes

**Fig. 3:**
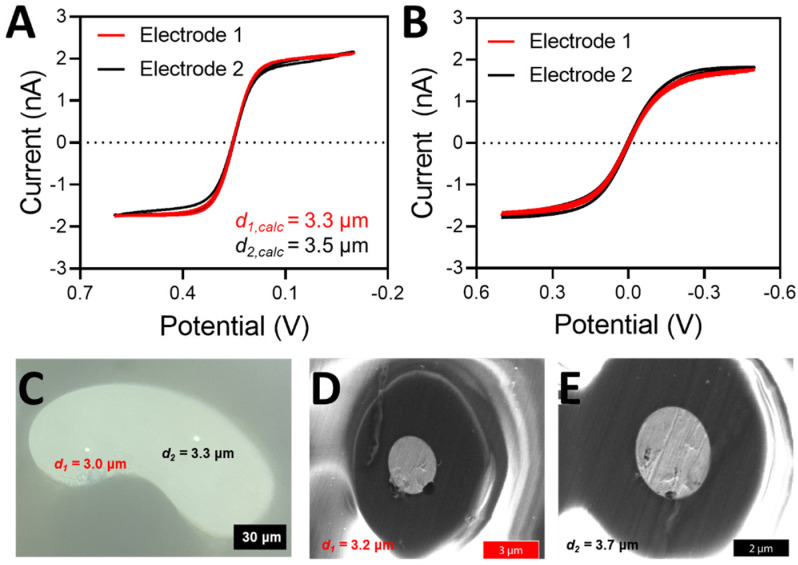
(A) Cyclic voltammetry in 5 mM potassium hexacyanoferrate(ii/iii) solution in 1 M KCl with electrode 1 (red) and electrode 2 (black) *versus* a true Ag/AgCl reference electrode with a Pt wire counter electrode. Voltammetry was taken from 0.6 to −0.1 V after 2 seconds of quiet time at a scan rate of 10 mV s^−1^ over 3 scans. (B) Cyclic voltammetry in 5 mM potassium hexacyanoferrate(ii/iii) solution in 1 M KCl with electrode 1 (red) and electrode 2 (black) *versus* the opposite electrode as a quasi-reference counter electrode. Voltammetry was taken from 0.5 to −0.5 V after 2 seconds of quiet time at a scan rate of 100 mV s^−1^ over 3 scans. (C) A microscope image of the fabricated electrode at 50× magnification. (D) and (E) SEM images of the electrodes depicting the quality of the electrode seal.

After the proper seal has been achieved, the electrodes have been pulled, and an electrical connection has been made (see Materials and Methods), the electrodes can be electrochemically characterized with cyclic voltammetry and microscopy. An example of a properly sealed DBE that demonstrated ideal voltammetric behavior can be seen in Fig. 3. The electrodes are tested both against a true reference electrode (here we used a CHI Ag/AgCl reference electrode) (Fig. 3A) and the opposite electrode in the DBE (Fig. 3B). The limiting current from voltammetry taken against a true reference electrode can be used to calculate the theoretical electrode radius (eqn (S1)†). For a properly fabricated and functional DBE, the size that results from the limiting current calculations of each method should agree closely. If they do not agree, it is likely that more of the wire than an inlaid disk is exposed (*i.e.* excess wire needs to be beveled down or the seal is incomplete). This can be confirmed *via* microscopy as discussed below.

The size of the electrode determined from voltammetry should always be compared to a measured value obtained from microscopy (Fig. 3C). If the electrode disks are large enough, they can be visualized and measured with a simple benchtop microscope. However, if they are smaller than 1 μm in diameter, scanning electron microscopy (SEM) can be used to visualize the electrode surfaces, as well as the state of the seal of the electrodes (Fig. 3D and E). An SEM image of the entire electrode surface can be found in Fig. S2.† Microscopy is a vital component to electrode characterization, because many errors can occur during electrode fabrication. For instance, the Pt disk can be recessed below the glass insulating material; the seal between the Pt wire and the glass may be imperfect; or there may be Pt wire protruding above the glass portion. These issues may affect the voltammetry in specific ways while remaining unnoticed. For instance, the steady state current of a recessed electrode may look ideal, but it is likely indicating a smaller-than-actual radius because the redox species are undergoing linear diffusion instead of radial diffusion.^26^ A gap in the seal can expose the sides of the Pt wire to the redox species, significantly altering the voltammetry.^28^ If the wire is protruding above the glass, it no longer has the ideal, disk shaped geometry, and thus we can no longer use the analytical solution *i* = 4*nFDCr* to accurately determine the electrode radius. Thus, whenever possible, microscopy should be used to confirm the electrode geometry and size. Here, example SEM images of failed DBE fabrication can be seen in Fig. S3.† Determination of the electrode size is easier to accomplish with a high magnification on a standing optical microscope when the electrodes are large enough to be resolved, but SEM images will often provide better insight into the quality of seal. However, when using SEM for the imaging of the DBEs, proper grounding of the electrode is crucial to maintaining electrode function. The measured electrode diameters from both presented methods are within 10% of one another, and within 10% of the value calculated by the limiting current.

**Fig. 4:**
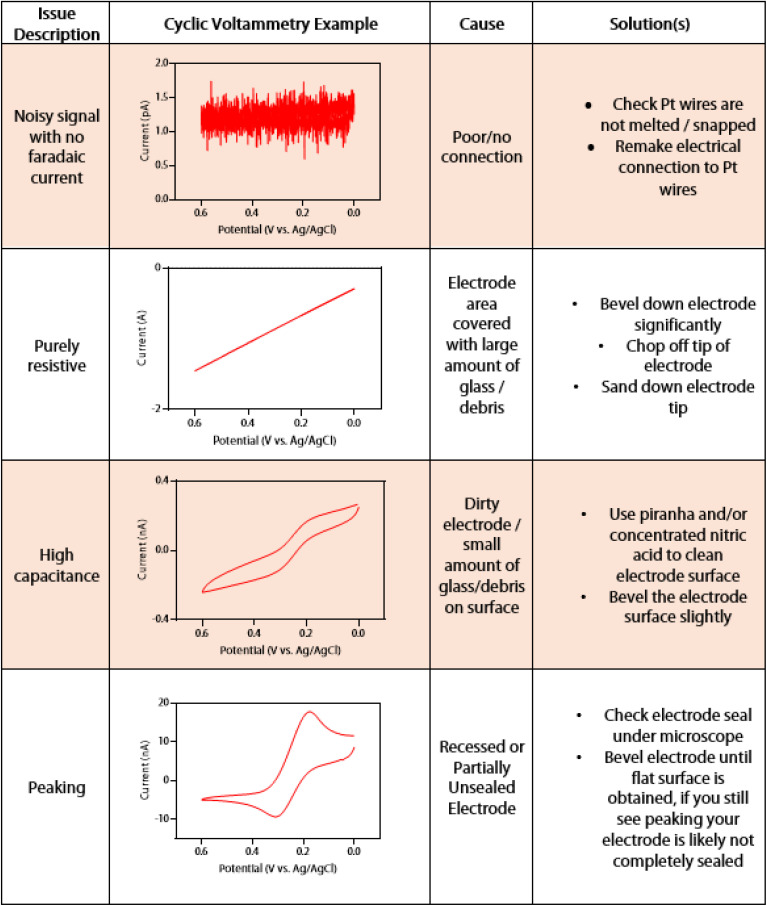
DBE cyclic voltammetry troubleshooting guide. All voltammetry was taken with one electrode of a DBE *vs.* an Ag/AgCl reference electrode with a Pt wire counter electrode from 0 to 0.6 V with a scan rate of 0.01 V s^−1^ and a quiet time of 2 seconds.

If non-ideal voltammetry is seen from the dual barrel electrode, the issue can be diagnosed according to the cyclic voltammetry troubleshooting guide in Fig. 4. A quick method to determine if there is connection between each electrode is to test them against one another. If the signal is noisy and no faradaic current can be seen across the potential window, there is no connection between the potentiostat and the electrode tip where the reaction should be happening. In this case, the electrical connection may need to be remade between the sealed platinum wire and the gallium-dipped electrical wire. If the issue persists, and the Pt wires are not snapped or melted, there is likely a disconnect within the sealed portion of the Pt wire that cannot be observed from microscopy. If the signal is a linear slant, it is most likely that the electrode is still covered with a large amount of glass or debris. It may also be that there is a leak or partially open electrode, as this behavior mimics that of an open pipette.^29,30^ In this case, the electrode should be carefully beveled down until a faradaic current is observed. This may take a significant amount of beveling, but with proper care, a faradaic current should begin to be seen. The signal may initially be highly capacitive. When the faradaic current shows high capacitance, the electrode has only a small amount of debris on the surface. A cleaning solution (such as piranha or nitric acid) can be used to chemically remove the debris, or the electrode can be very briefly polished *via* a micropipette beveler to mechanically remove the rest of the debris. Another indication of an electrode that needs to be polished is if the voltammetry shows peaking behavior. In this case, the electrode does not show a sigmoidal shape but the more classic “duck” shape. The electrode is either recessed or partially unsealed, and it must be polished down to a sealed, inlaid disk. As an unsealed portion of the disk is nearly impossible to see under normal benchtop microscopy, SEM may be needed to visualize this issue. If one does not have access to SEM, the electrode can continue to be polished until the expected sigmoidal shape of the current trace is seen. Examples of how beveling a dual barrel electrode can restore its sigmoidal shape and remove capacitance from the voltammetry can be seen in Fig. S4.†

**Fig. 5:**
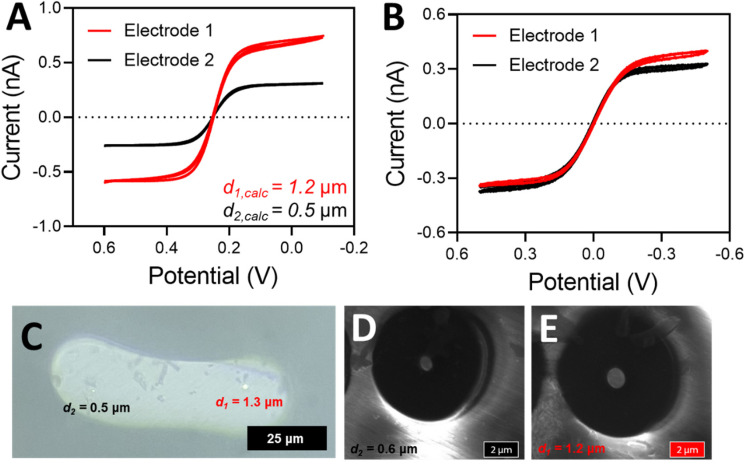
(A) Cyclic voltammetry in 5 mM potassium hexacyanoferrate(ii/iii) solution in 1 M KCl with electrode 1 (red) and electrode 2 (black) *versus* a true Ag/AgCl reference electrode with a Pt wire counter electrode. Voltammetry was taken from 0.6 to −0.1 V after 2 seconds of quiet time at a scan rate of 10 mV s^−1^ over 3 scans. (B) Cyclic voltammetry in 5 mM potassium hexacyanoferrate(ii/iii) solution in 1 M KCl with electrode 1 (red) and electrode 2 (black) *versus* the opposite electrode as a quasi-reference counter electrode. Voltammetry was taken from 0.5 to −0.5 V after 2 seconds of quiet time at a scan rate of 100 mV s^−1^ over 3 scans. (C) A microscope image of the fabricated electrode at 50× magnification. (D) and (E) SEM images of the electrodes.

Performing voltammetry against a true reference is ultimately critical, as the observed current when referencing against a DBE is limited by the smallest electrode (regardless of which electrode is being used as the working or the quasi-reference counter electrode). The current being passed at the working electrode will be limited if the counter electrode cannot pass the same amount of current (because it is smaller than the working electrode, for example). Thus, if the counter electrode is smaller than the working electrode, the limiting current of the voltammogram is more indicative of what is happening at the counter electrode rather than at the working electrode. An example of this scenario can be seen in Fig. 5. As shown in Fig. 5B, the electrode sizes based on the limiting current appear to be very similar when one platinum disk is referenced against the other disk. However, when each individual disk is referenced against a true Ag/AgCl reference electrode (Fig. 5A), it becomes apparent that the disk sizes are significantly different. According to this voltammetry, electrode 1 is more than twice as large as electrode 2. Fig. 5A is in fact the true radius of the electrodes, as microscopy measurements closely match the electrode radius determined from the voltammetry with a true reference electrode (Fig. 5C–E). In contrast, the two voltammograms in Fig. 5B are both effectively reporting the size of the smaller electrode, regardless of if it is connected as the working or the counter electrode. The smaller electrode limits the amount of current passing through the system.^27^ Thus, electrochemical determination of the electrode radius should be confirmed with microscopy. An SEM image of the entire electrode surface can be found in Fig. S5.† In agreement with the electrode presented in Fig. 3, the optical and SEM images agree within 10% with one another as well as with the limiting current from voltammetry (in the case of a true reference being used). It is also important to note that the electrode used in Fig. 3 does not suffer from the same limitation as we have discussed here because the electrode sizes are so similar.

### Experimental considerations

**Fig. 6:**
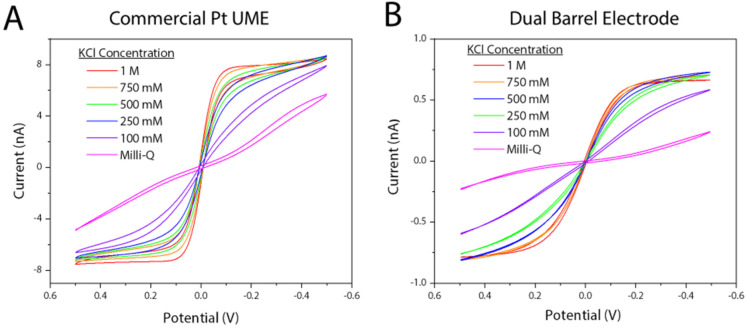
Changes in voltammetry with changing salt concentration on a (A) commercial CHI platinum UME (*r* = 5 μm) working electrode with a Pt wire quasi-reference counter electrode and (B) a dual barrel electrode (WE *r* = 0.59 μm, QRCE *r* = 0.60 μm). Voltammetry was taken in a 5 mM potassium hexacyanoferrate(ii/iii) solution with changing salt concentration from 0.5 V to −0.5 V at a scan rate of 0.1 V s^−1^ with a 2 seconds quiet time for 5 scans. The final scan for each voltammogram is plotted in polarographic convention.

To show the DBE acts similarly to a commercial CHI Pt microelectrode (when a platinum wire is used as a quasi-reference counter electrode), both were tested in 5 mM potassium hexacyanoferrate(ii/iii) solutions with varying salt concentrations. As seen in Fig. 6, the DBE yields similar voltammetry in all tested salt concentrations. It is important to note that using the DBE electrodes for the working and quasi-reference electrodes can cause shifting in expected *E*_1/2_ values, especially in solutions where only either the oxidized or reduced form is present. Here, we have shown voltammetry in solutions containing equal concentrations of both the oxidized and reduced forms of a well-behaved redox mediator, and as such, all observed *E*_1/2_ values have been around 0 V. As oxidation of hexacyanoferrate(ii) occurs at the working electrode, the reverse reaction (*i.e.*, reduction of hexacyanoferrate(iii)) is happening at the quasi-reference/counter electrode. Simulations in previous work using the same electrode and redox mediator system show that the diffusion layers do not overlap,^20^ and so there is no competing flux from the two discs. Shifting of *E*_1/2_ values with a quasi-reference is observed with only one form of the redox mediator present when a UME is referenced against a large quasi-reference counter electrode (Fig. S6†). However, when a DBE is used and referenced against itself, the observed shift can be large, where a potential shift of 700 mV was observed when only the oxidized form (potassium hexacyanoferrate(iii)) was present in solution (Fig. S7†). This observed potential shift also occurs when using two commercially available Pt UMEs, one as the working electrode and the other as the quasi-reference/counter electrode (Fig. S8†). When using the DBEs for a specific mediator, a large window should be used to find the observed *E*_1/2_ value to account for possible large potential shifts.

When performing voltammetry with the DBEs, it is important to keep the electrodes from discharging. Nioradze *et al.* previously published a work detailing the effect of electrostatic discharge on nano and micrometer size electrodes, demonstrating the importance of preventing such a discharge.^31^ From our experiments, we have observed discharging when the potentiostat cell turns off and on between runs. Discharging of the electrode can irreversibly compromise the ability to take measurements with the electrode (Fig. S9†). This can even happen in instances of two commercial CHI UMEs being used as working and quasi-reference counter electrodes. For CHI potentiostats (which were used here-in) it is crucial to keep the “cell on between runs” function enabled while using the dual barrel electrodes to prevent this. When this function is enabled, no such behavior is observed. It should also be noted that the DBEs have never demonstrated a discharge effect in high salt concentrations (1 M KCl) even with this function disabled, and as such, that was the KCl concentration used for all voltammetry presented.

## Conclusion

Dual barrel electrodes can be a powerful tool for electroanalysis. Because there are many things that can go wrong with a DBE, a new user must consider both the fabrication parameters as well as the experimental conditions. Importantly, we have demonstrated how significant it is to have the correct heat and number of sealing cycles to avoid wire melting or snapping. Once fabricated, the electrodes should be beveled and characterized so that the voltammetry and microscopy measurements agree. The experimental considerations are equally important, as large potential shifts can be observed when one electrode is referenced against the other. Our goal with this paper is to enhance reproducibility in the development of new electrode probes that have a myriad of applications.

## Materials and methods

### Reagents and materials

Potassium chloride (KCl) was purchased from Thermo Fisher Scientific. Potassium ferrocyanide trihydrate (99+% for analysis) and potassium ferricyanide (99+%, ACS reagent) were purchased from Acros Organics. Gallium (99.9% trace metal basis) was purchased from Sigma Aldrich. All chemicals were used as received. Aqueous solutions were prepared with ultrapure water (Millipore Milli-Q, 18.2 MΩ cm). An Ultrasonic Cleaner with Digital Timer (VWR, Radnor, Pennsylvania) was used to sonicate all solutions. All platinum wires [25 μm and 250 μm] (for DBE fabrication, larger UME fabrication, and use as a quasi-reference counter electrode) were purchased from Alfa Aesar. 22AWG silicone hook up wire (OD: 1.7 mm) – 22 gauge stranded tinned copper wire with silicone insulation, 6 colors (black, red, yellow, green, blue, white) 23 ft/7 m each, hook up wire kit from Plusivo was used for making electrical connection in the DBEs. Quartz theta glass capillaries (1.20/0.9 mm OD/ID) and a P-2000 laser-based micropipette puller (Sutter Instrument, Novato, California) were used for DBE fabrication. A BV-10 Microelectrode Beveler with an extra fine (0.2 to 1.0 μm tip sizes) diamond abrasive plate (Sutter Instrument, Novato, California) was used for beveling of DBEs. A rotary vacuum pump (RZ 6) was purchased from Vacuubrand. Vacuum tubes were purchased from Fisher Scientifics (60985-540, 14-469-1A) and New Age Industries (1400154). A Leica DM750 Binocular Upright Microscope was used to monitor the electrode seal and surface. All microscope images used in this publication were taken with a personal smartphone, iPhone 12 Pro Max, by aligning the phone with an eyepiece of the microscope. Electrochemical experiments were performed using a CHI model 601E or 601D potentiostat (CH Instruments, Austin, Texas) with a three-electrode cell placed in a Faraday cage. A platinum ultramicroelectrode (UME) [*r* = 5 μm] (CH Instruments, Austin, Texas) and an Ag/AgCl (CH Instruments, Austin, Texas) reference electrode were used for control experiments. A FEI Quanta 3D FEG dual-beam SEM with an Oxford INCA Xstream-2 silicon drift detector was used for SEM and EDX imaging.

### Dual barrel electrode (DBE) fabrication

A small length (3–6 cm) of Pt wire [*d* = 25 μm] was threaded into each channel of a quartz theta glass capillary. The capillary was then tapped lightly on a hard surface until the Pt wire reached the middle of the capillary. The laser puller was turned on 15 minutes before securing the threaded capillary in place with metal clamps and a metal insert (preventing the clamps from pulling apart). A vacuum line was attached to each end of the capillary and the vacuum was allowed to run for 2 minutes before the sealing process. The capillary was then sealed with several cycles of 30 seconds with the laser on followed by 30 seconds of the laser off. The capillary was rotated 180 degrees halfway through the full number of cycles in the sealing process. After sealing, the metal inserts and vacuum line were removed before the pull step was initiated. After the pull step, the pulled electrodes were removed from the puller. Electrical wire was cut and stripped to the length of the glass capillary. The electrical wire was then dipped in gallium (which is stored at 55 °C to ensure it remains a liquid) and threaded into a barrel of the dual barrel electrode with a small length still outside of the capillary (about 1–2 cm). This process was repeated with electrical wire of a different color to make connection with the other electrode in the other barrel. The wires were secured into place with hot glue, leaving a small length of wire still exposed. Copper tape was secured around the exposed wire to allow for better connection with the electrode leads from the potentiostat. The electrode was then used to run cyclic voltammetry in a 5 mM potassium hexacyanoferrate(ii/iii) in 1 M KCl solution. The electrode was beveled and cleaned as needed until a clean CV was obtained.

## Author contributions

L. E. K., P. J. K., and A. K. W. performed all experiments. P. J. K. and L. E. K. wrote the manuscript. J. E. D. supervised all aspects of the research presented. All authors have given approval to the final version of the manuscript.

## Conflicts of interest

There are no conflicts of interest to declare.

## Supplementary Material

AN-149-D3AN01969A-s001
