# Supplementary material for: Considerations for dual barrel electrode fabrication and experimentation
Source: Analyst. 2024 Jan 23;149(7):2180–9. doi: 10.1039/d3an01969a (PMC10962018; doi:10.1039/d3an01969a)
Supplement: AN-149-D3AN01969A-s001 [file AN-149-D3AN01969A-s001.pdf]

Electronic Supplementary Material (ESI) for Analyst.
This journal is © The Royal Society of Chemistry 2024

Supporting Information for

Considerations for Dual Barrel Electrode Fabrication and Experimentation

Lynn E. Krushinski[a§], Philip J. Kauffmann[a§], Amber K. Wang[a], and Jeffrey E. Dick[a,b]*

[a]Department of Chemistry, Purdue University, West Lafayette, IN, 47907, USA

[b] Elmore Family School of Electrical and Computer Engineering, Purdue University, West Lafayette, IN 47907, USA

[§]Indicates Equal Contribution

To whom correspondence should be addressed: jdick@purdue.edu

Table of Contents

Step by step laser pulling guide (Figure S1) S2
Calculation of Electrode Area from Limiting Current S3
SEM Image of Dual Barrel Electrode from **Figure 3** (Figure S2) S4
SEM Images of Failed DBE Fabrication (Figure S3) S5
Effect of Properly Beveling Dual Barrel Electrodes (Figure S4) S6
SEM Image of Dual Barrel Electrode from **Figure 5** (Figure S5) S7
Voltammetry of solutions containing Fi, Fo, and Fi/Fo on a commercial UME (Figure S6)...... S8
Changes in Half-Wave Potential With changing reference electrode (Figure S7) S9
Potential Change With Commercial Pt UMEs and a Single Redox Molecule (Figure S8)…….S10
Effect of Discharge at the Electrode on the Cyclic Voltammetry (Figure S9) S11

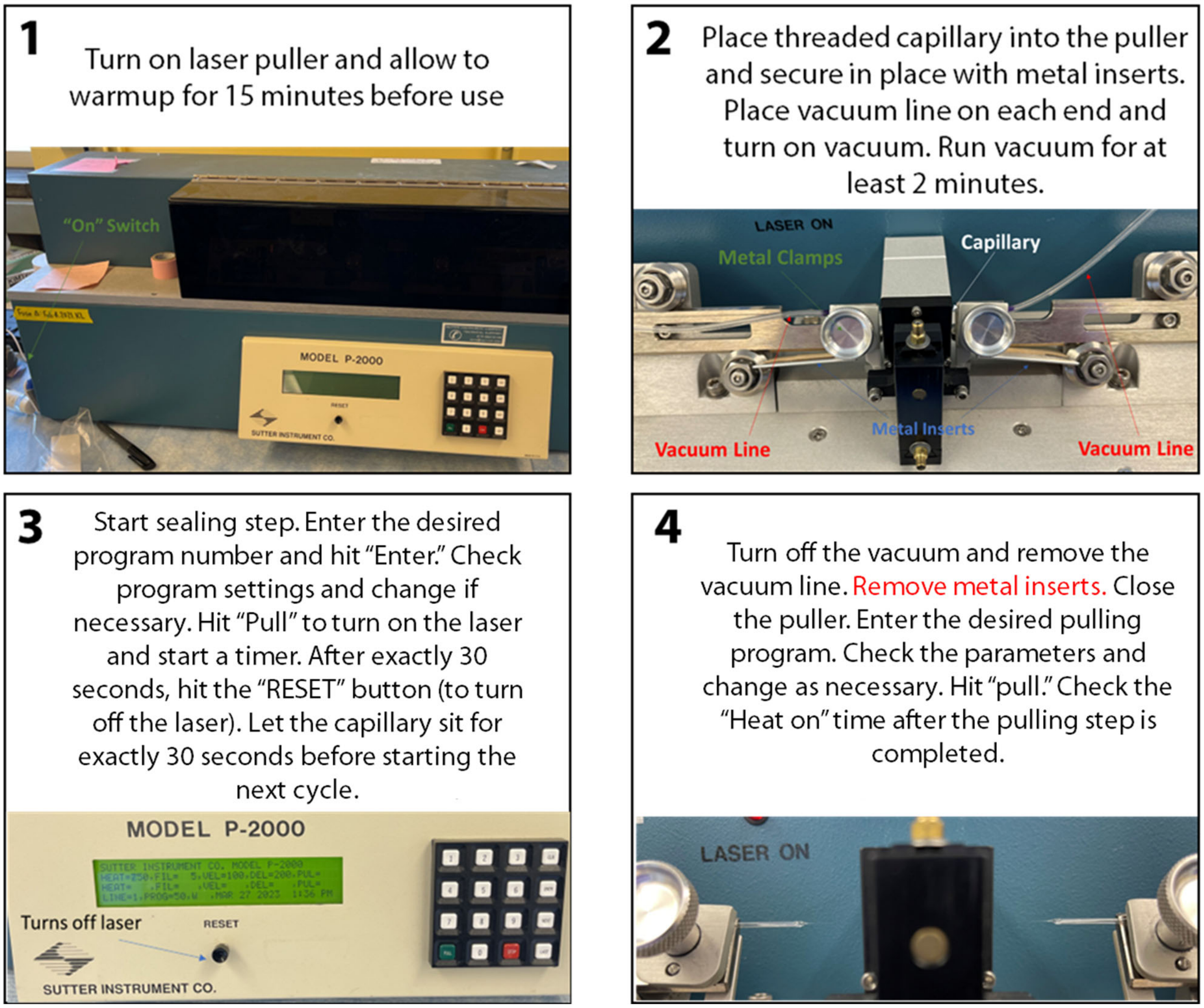

Figure S1. Step by step dual barrel electrode (DBE) laser pulling procedure.

Equation S1: Calculation of Electrode Area from Limiting Current

The electrode radius can be calculated using the equation $i_{ss} = 4nFDCr$, where i_{ss} is the steady state current, n is the stoichiometric number of electrons, F is Faraday's constant (96485 C•mol^{-1}), D is the diffusion coefficient of redox species (6.5 x 10^{-10} m^2•s^{-1} for potassium hexacyanoferrate (II/III)), C is the concentration of the redox species (5 mM), and r is the radius of the electrode (the value we calculate). Thus, for a cyclic voltammogram with a limiting current of -1.72 x 10^{-9} A (from **Figure 3A**, Electrode 1 and 2 Anodic Limiting Current):

Electrode 1: (1.72 x 10^{-9} A) = (4)(1)(96485 C•mol^{-1})(6.5 x 10^{-10} m^2•s^{-1})*(5 mM)*($\boldsymbol{r_1}$)

$\boldsymbol{r_1}$ = 1.37 µm radius

Electrode 2: (1.72 x 10^{-9} A) = (4)(1)(96485 C•mol^{-1})(6.5 x 10^{-10} m^2•s^{-1})*(5 mM)*($\boldsymbol{r_2}$)

$\boldsymbol{r_2}$ = 1.40 µm radius

Disk diameter from optical microscopy(d_{meas}):

Electrode 1: 3.0 µm

Electrode 2: 3.3 µm

Percent Difference:

Electrode 1: [(2*r_1)- d_{meas}]/ d_{meas} *100 = 8.6%

Electrode 2: [(2*r_2)- d_{meas}]/ d_{meas}*100 = 15%

It should be noted that the anodic limiting current from the oxidation of potassium hexacyanoferrate (III) was chosen for calculation of the theoretical radius as to avoid the effect oxygen may have on the cathodic limiting current.

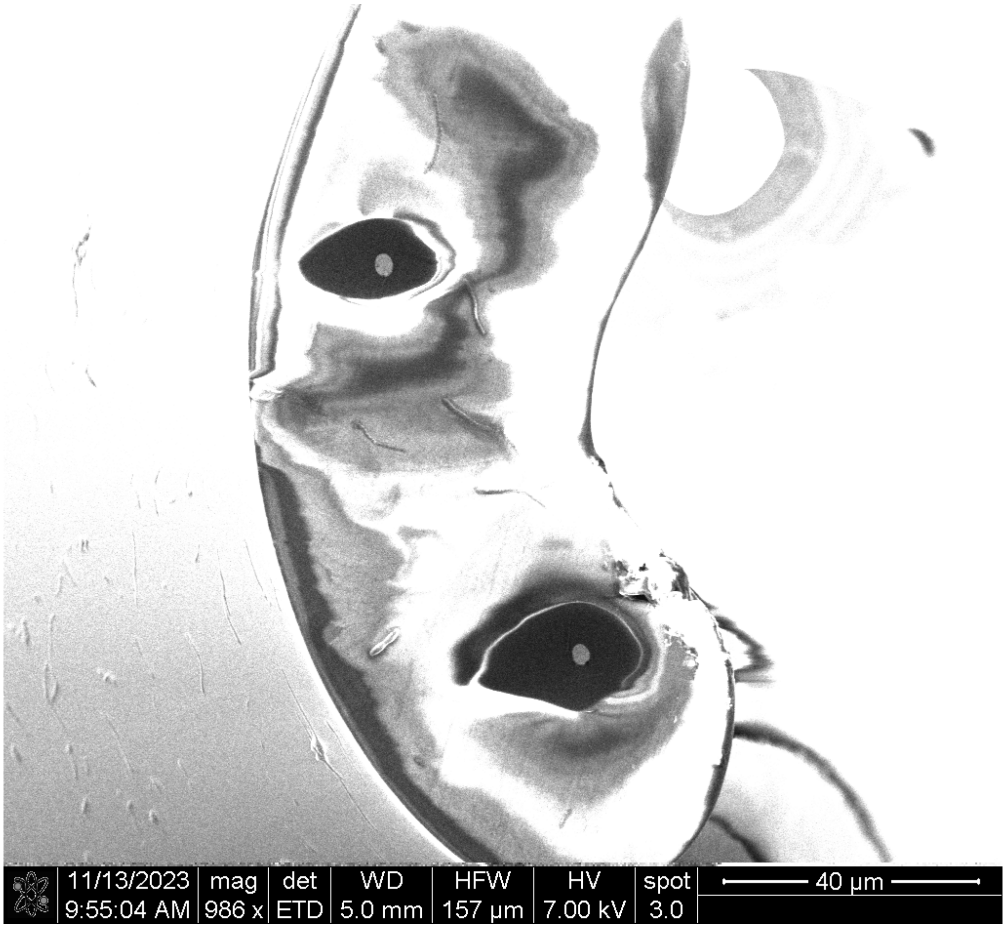

Figure S2. SEM image of the entire surface of the DBE presented in **Figure 2.**

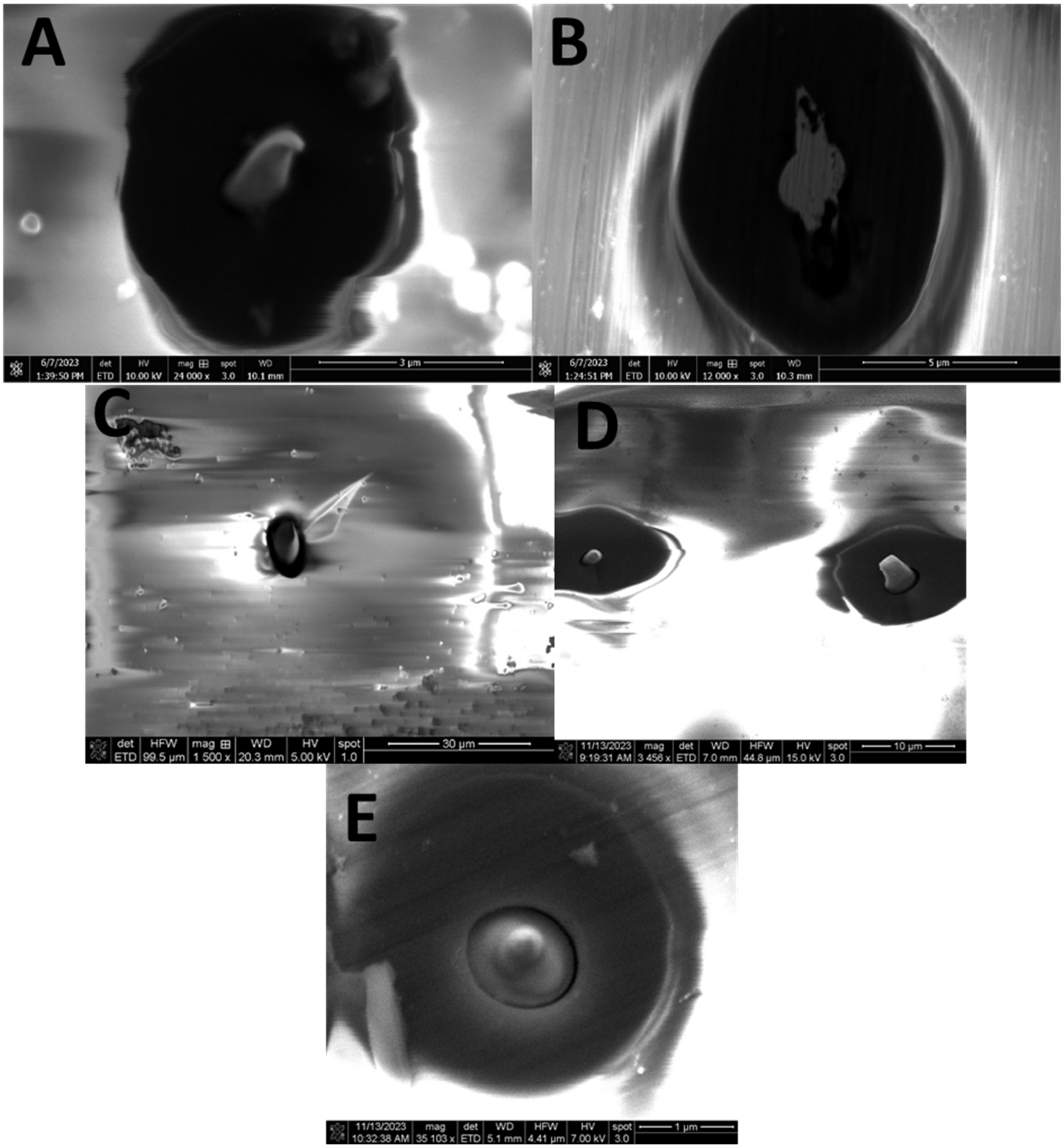

Figure S3. SEM images of failed dual barrel electrode fabrication. In **A**, there is a portion of wire sticking out of the sealed glass. To fix this, the DBE needs to be carefully beveled to remove this excess wire. In **B**, the wire is sealed, but misshapen, indicating that the wire melted slightly too much during the sealing step. In **C** and **D**, the glass does not encase and seal the wire, indicating that more cycles or higher heat settings should be used next time. In **E**, the electrode is partially recessed. This occurred as a result of improper grounding during SEM imaging.

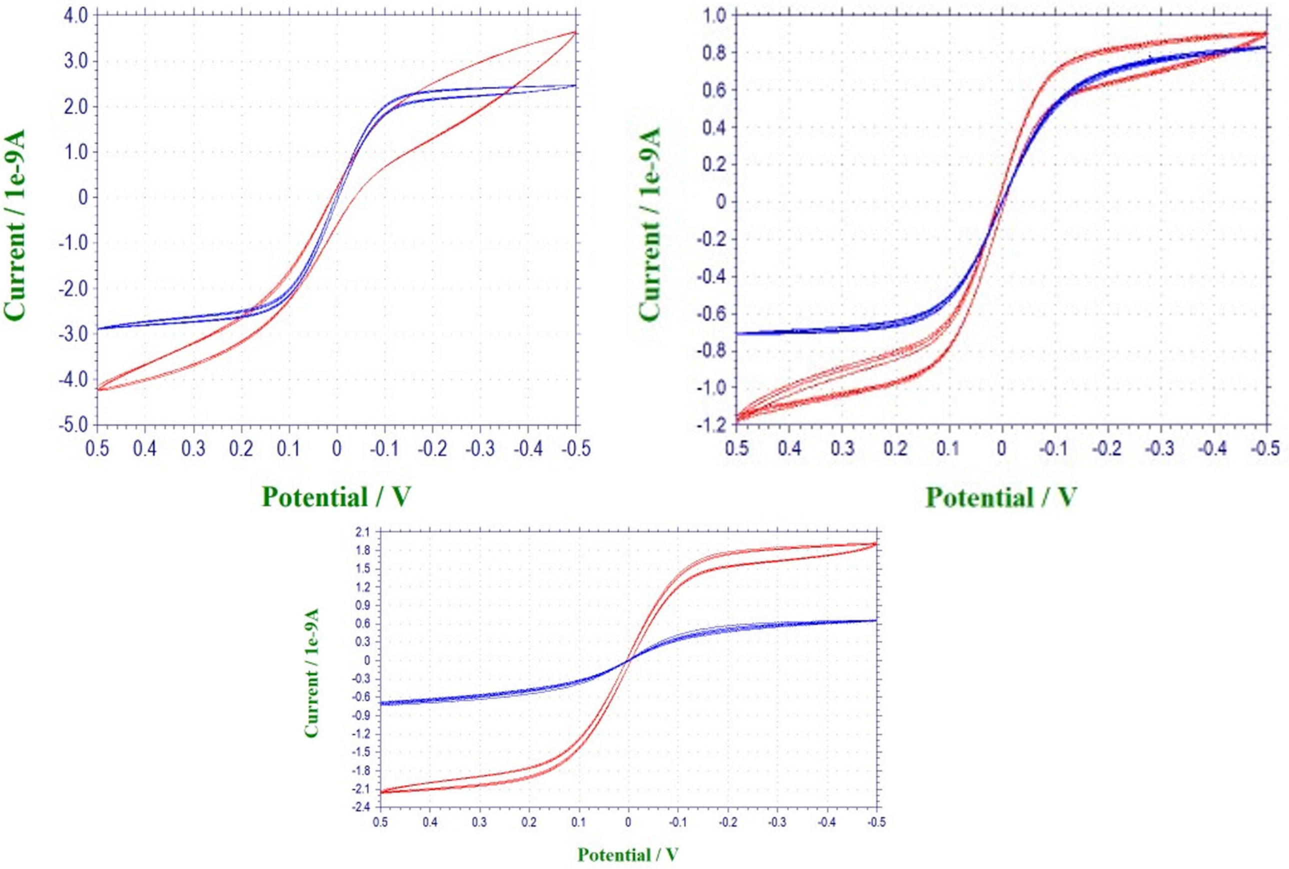

Figure S4. Overlaid voltammetry of three different DBEs in 5 mM potassium ferrocyanide and 5 mM potassium ferricyanide in 1 M KCl with the same working electrode in a dual barrel before (red) and after (blue) beveling. Voltammetry was taken from 0.5 to -0.5 V at a scan rate of 100 mV/s over 3 scans after a quiet time of 2 seconds with the other electrode in the dual barrel as the quasi-reference counter electrode.

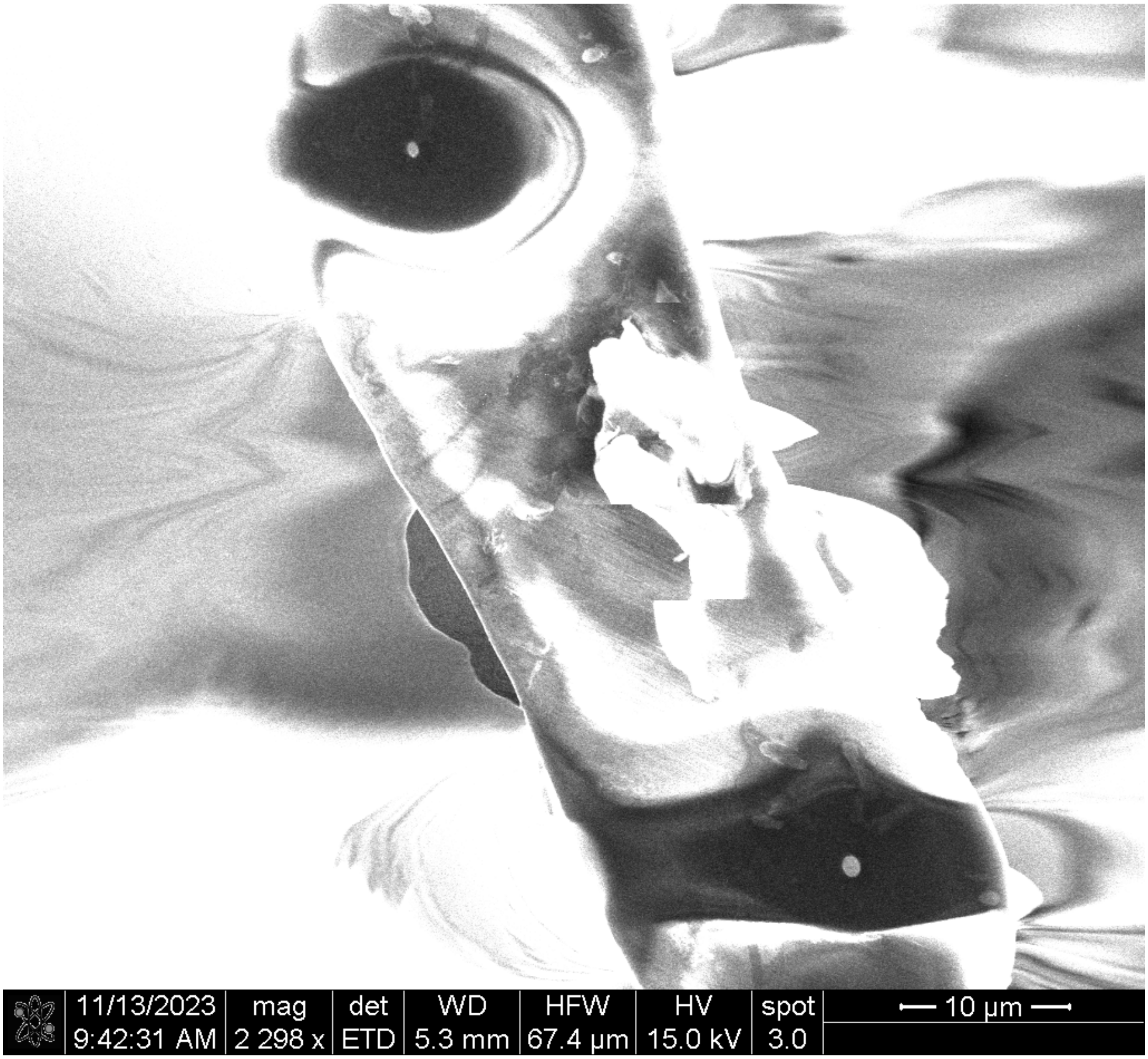

Figure S5. SEM image of the entire surface of the DBE presented in **Figure 4.**

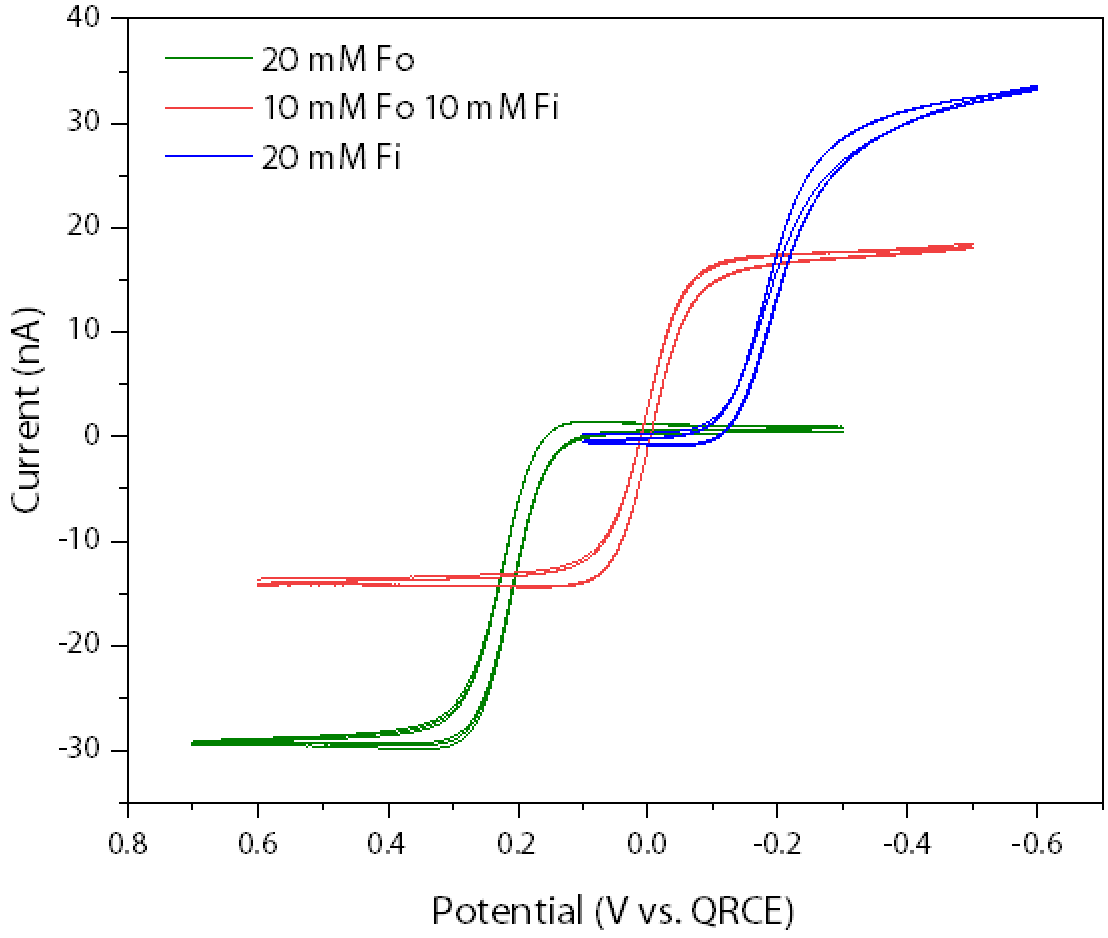

Figure S6. Cyclic voltammetry of 20 mM potassium hexacyanoferrate (III) (green), 10 mM potassium hexacyanoferrate (II/III) (red), and 20 mM potassium hexacyanoferrate (II) (blue) in 1 M KCl. Voltammograms were taken with a CHI Pt UME (r = 5 µm) working electrode and a Pt wire quasi-reference counter electrode at a scan rate of 0.1 V/s over three scans with a quiet time of two seconds.

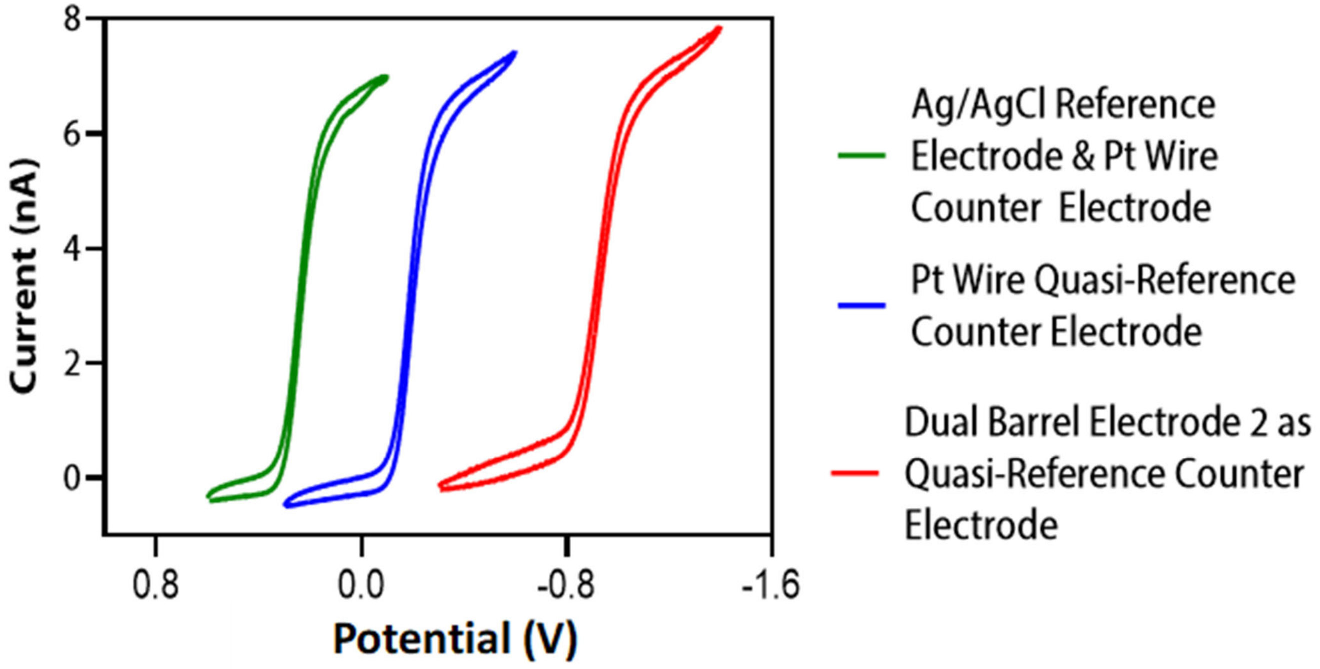

Figure S7. Changes in half-wave potential with changing reference electrode. The same working electrode (Electrode 1 on a DBE) was used for all measurements in a 5 mM potassium hexacyanoferrate (II) solution in 1 M KCl. Voltammetry was taken at a scan rate of 0.1 V/s with a 2 second quiet time and is plotted in polarographic convention.

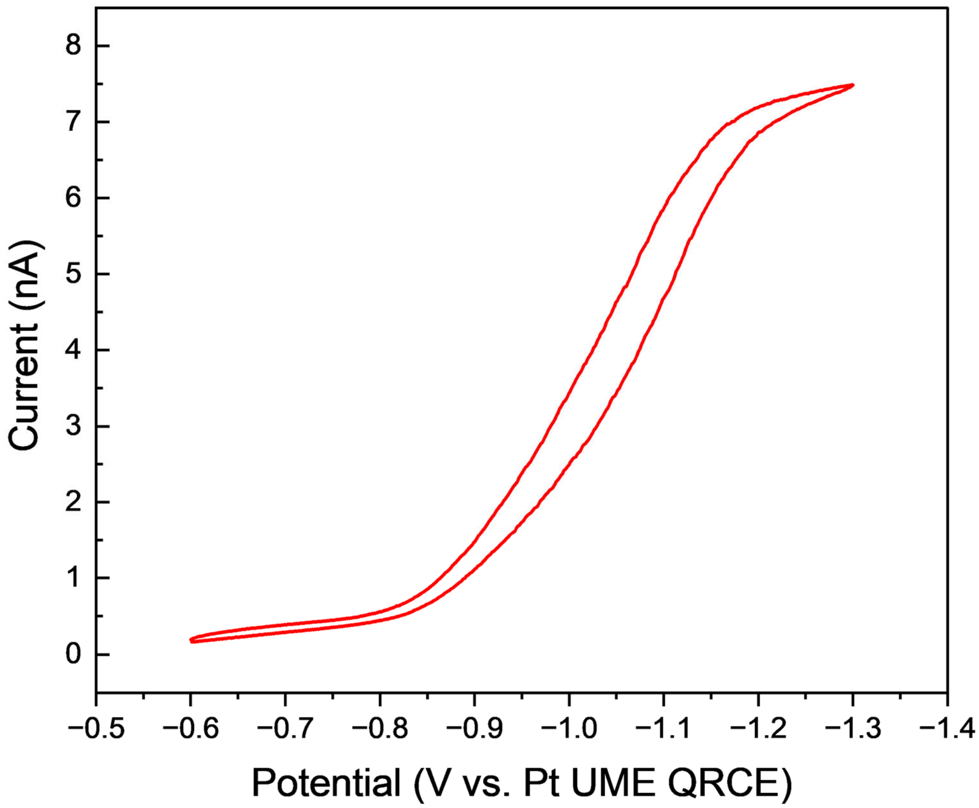

Figure S8. The cyclic voltammogram of 5mM potassium hexacyanoferrate (II) in 1M KCl, using commercial Pt UMEs (from CHI) as the working electrode and the quasi-reference/counter electrode. Voltammetry was taken at a scan rate of 0.1 V/s with no quiet time and is plotted in Polarographic convention. This potential shift matches the shift seen with the dual barrel electrode in similar experimental conditions.

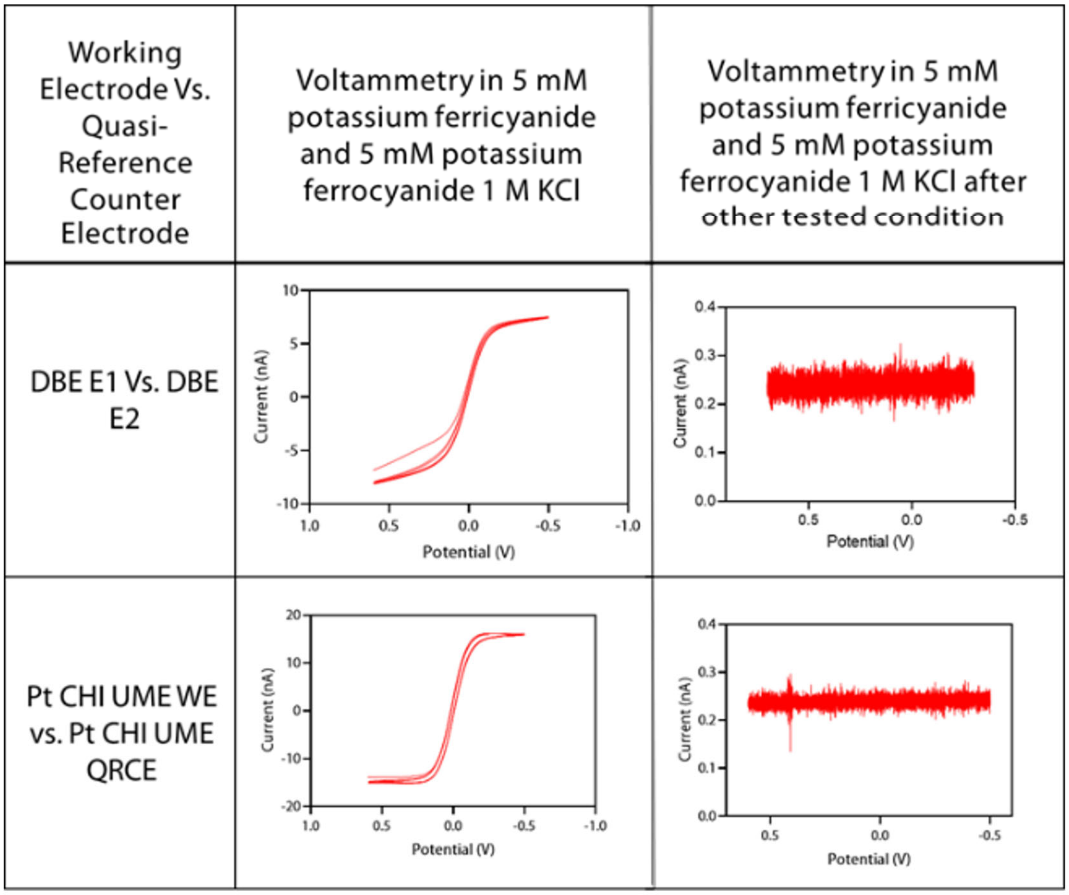

Figure S9. Examples of voltammetry after a discharge on both a dual barrel electrode (with one electrode as working and another as a quasi-reference counter) and a mimic system using two CHI Pt UMEs (r = 5 µm). It should be noted that only discharge in a 10 mM potassium ferrocyanide system is presented, but similar effects have been seen in solutions of only potassium ferricyanide and solutions with salt concentrations below 750 mM.
